# Group I Intron Internal Guide Sequence Binding Strength as a Component of Ribozyme Network Formation

**DOI:** 10.3390/molecules21101293

**Published:** 2016-09-27

**Authors:** Laura Elizabeth Satterwhite, Jessica A. M. Yeates, Niles Lehman

**Affiliations:** Department of Chemistry, Portland State University, Portland, OR 97202, USA; satter2@pdx.edu (L.E.S.); jmellor@pdx.edu (J.A.M.Y.)

**Keywords:** RNA, ribozymes, origins of life, internal guide sequence, recombination, fluorescence anisotropy

## Abstract

Origins-of-life research requires searching for a plausible transition from simple chemicals to larger macromolecules that can both hold information and catalyze their own production. We have previously shown that some group I intron ribozymes possess the ability to help synthesize other ribozyme genotypes by recombination reactions in small networks in an autocatalytic fashion. By simplifying these recombination reactions, using fluorescent anisotropy, we quantified the thermodynamic binding strength between two nucleotides of two group I intron RNA fragments for all 16 possible genotype combinations. We provide evidence that the binding strength (*K*_D_) between the 3-nucleotide internal guide sequence (IGS) of one ribozyme and its complement in another is correlated to the catalytic ability of the ribozyme. This work demonstrates that one can begin to deconstruct the thermodynamic basis of information in prebiotic RNA systems.

## 1. Introduction

A key problem in origins-of-life research is the advent of informational complexity. The “RNA world” hypothesis posits that the dual capacity of RNA molecules to store information and provide catalytic potential makes this molecule a good candidate for an early pre-life driving force [1]. However, under such a scenario there needs to be a mechanism for small abiotic RNA oligomers to grow in size and be subject to a primitive selective process. Previously, we have shown that fragments of the group I intron derived from the isoleucine tRNA from the purple bacterium *Azoarcus* can act as a general RNA recombinase [2]. This feature led to the discoveries that the ribozyme can self-assemble from its own fragments [3], that cooperative RNA networks could form from pools of variant fragments [4], and that a game-theoretic analysis can partially explain how 2- and 3-membered such networks can form and change composition over time [5]. It is unclear whether this specific type of information transfer was used in RNA world pre-life systems. However, several concepts relevant to the emergence of function from a pool of sequences can be studied with this model system, thereby probing key steps in the mergence of an RNA world.

**Figure 1:**
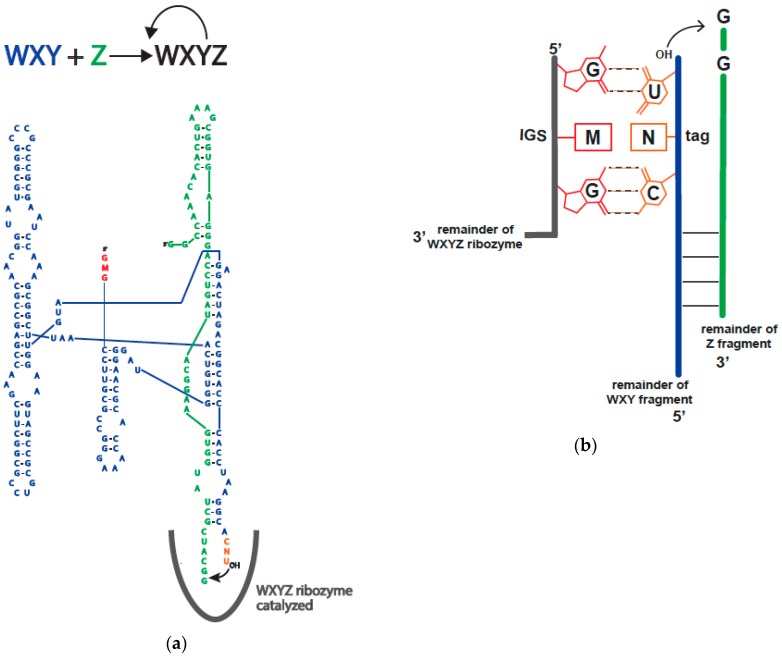
The covalent self-assembly reaction by fragments of the *Azoarcus* group I intron ribozyme. (**a**) A schematic of the **WXY** (155 nt) + **Z** (45 nt) autocatalytic reaction, as first described in ref. [3]. The internal guide sequence (IGS) (red) of a **WXYZ** ribozyme (either in pieces or covalently contiguous) binds to the tag (orange) of a **WXY** fragment (blue) and catalyzes a recombination reaction with a **Z** fragment (green) to produce another **WXYZ** ribozyme, plus a free guanosine nucleotide. The reaction is autocatalytic; (**b**) A blowup of the IGS-tag interaction, where the middle nucleotides of the IGS (i.e., M) and of the tag (i.e., N) can be freely varied to all 16 possible combinations [5].

The ability of recombinase ribozymes to exhibit such a variety of activity is due to only a 1- or 2-nucleotide (nt) change within the internal guide sequence (IGS) and/or its complementary “tag” sequences (Figure 1). Based on catalytic prowess, we were able to predict and engineer specific multi-interacting RNA population outcomes simply from the autocatalytic rate constants of these various IGS/tag sequences [5]. Despite the significance of this nucleotide-pair triplet, the underlying contribution of this interaction is not fully understood, and yet it is precisely this thermodynamic interaction that forms the basis for the intermolecular (and interpopulation) behavior described in previous studies [2,3,4,5]. Thus in this paper we will investigate more thoroughly a critical facet of this triplet interaction: the binding strength, or *K*_D_ parameter.

During natural cellular self-splicing of the *Azoarcus* tRNA^Ile^ intron, the triplet IGS and tag recognition must be accurately selected from among many possible such nucleotide triplets. Failure to do so would result in insertions or deletions in the 5′ and 3’ splice sites of the tRNA precursor product. For group I introns in general, the two-step splicing reactions required to remove the intron and attach the exons utilize secondary interactions in the alignment of the G•U wobble splice site at the IGS-tag interface, as well as tertiary elements such as correct folding of the intron and positioning of an exogenous guanosine in the G-binding site [6,7,8,9]. The structural and sequence requirements of the IGS of group I introns has been studied in depth, particularly for the *Tetrahymena* ribozyme [10]. Analyses of the *Azoarcus*-ribozyme specific reaction show many similarities to that of the *Tetrahymena* ribozyme. Yet a focus on the 3-nt IGS of *Azoarcus* (compared to 6-nt in the *Tetrahymena* IGS) has revealed that the catalytic step is rate limiting in *Azoarcus* reaction [11] and that there is a greater amount of binding energy in the tertiary structure of the *Azoarcus* ribozyme, perhaps as compensation for the short length of the IGS [12]. These data underscore the critical nature of the thermodynamic contribution of each nucleotide pair in the IGS-tag interaction in this reaction. Moreover, it has been shown that the splicing of the *Azoarcus* tRNA is strongly dependent on the local secondary structure around the splice site [13], further underscoring the need to better understand the relationship between IGS sequence and binding flexibility.

The in vitro recombinase system, as in the canonical tRNA splicing reaction, would also need to utilize these secondary and tertiary elements to perform catalysis. The difference between the two situations revolves primarily around the condition that in vivo the ribozyme is performing an *intramolecular* reaction, where here, the recombinase performs an *intermolecular* reaction. Our mechanism primarily proceeds through a one-step process (Figure 1) [14], noticed first by Zaug et al. [7], and is optimal at higher temperatures (e.g., 48 °C) and magnesium concentrations (e.g., 100 mM) than those found in a living cell.

Understanding the in vivo versus the in vitro designed ribozyme reaction allows us to consider the factors that could impart differential activity among genotypes. Given that only a 1- to 2-nucleotide mutation is performed at the IGS/tag locations (sites that are not known as tertiary structural elements; see ref. [15]), it is thought that the foundation for activity diversity lies within the secondary structural element of the IGS and tag recognition. In this work, we systematically varied the middle nucleotides of both the IGS and tag triplets, and measured the binding strengths of duplexes in a model system. We then correlated the resulting values to the known catalytic self-assembly rates in an attempt to dissect the contribution of single nucleotide pairs to the dynamics of prebiotic RNA network formation.

## 2. Results

### 2.1. Experimental Design

#### 2.1.1. Fluorescence Anisotropy

**Figure 2:**
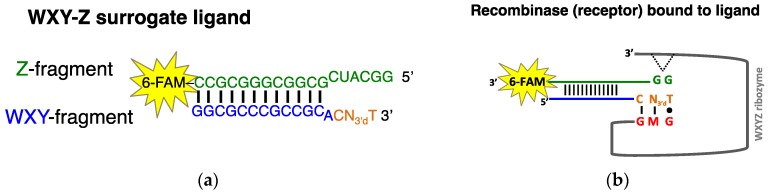
Fluorescence anisotropy ligand and receptor design. (**a**) Design of the surrogate **WXY**-**Z** ligand. The “**WXY fragment**” (blue) contains the tag (orange) and a 3′ deoxythymidine on the 3′ end prevents catalytic reaction. The “N” nucleotide represents an A, C, G, or C nucleotide. The **Z** fragment (green) contains the 3’ fluorescent label 6-FAM (6-carboxyfluorescein). The ligand molecular weight is approximately 11 kDa with a *T*_m_ = 90 °C; (**b**) Schematic of the surrogate **WXY**-**Z** ligand binding to the **WXYZ** recombinase ribozyme (receptor) at the IGS (red) and tag (orange) interface. A binding interaction also occurs between the G on the 5’ side of the “**Z fragment**” and the G-binding site on the ribozyme (indicated by dotted lines). The IGS “M” can represent any of the four nucleotides. The molecular mass of the *Azoarcus* recombinase ribozyme is approximately 64 kDa; see ref. [21] for complete sequence.

To investigate the thermodynamics of IGS-tag binding in the *Azoarcus* RNA covalent self-assembly reaction, we designed a model system in which RNA-RNA interactions could be detected by fluorescence anisotropy (FA) (Figure 2). We sought to isolate the IGS-tag binding interactions in a system in which the catalytic step cannot proceed, thus taking a different strategy from a standard kinetic approach. A direct binding assay, such as that provided by FA, is needed for the quantitation of the IGS-tag interaction. The **WXY** + **Z** ➜ **WXYZ** recombination reaction initiated by this triplet base-pair is autocatalytic and produces a continual increase in concentration of the ribozyme. While kinetic data can produce autocatalytic rate constants as described by von Kiedrowski [16,17], dissociation constants are not extractable as in a classic Michaelis-Menten analysis.

Fluorescence anisotropy is able to measure binding interactions by observing changes in the apparent size of a fluorescently labeled molecule. When a small fluorescent molecule is excited with plane-polarized light, the emitted light is depolarized due to rapid tumbling in solution during its fluorescence lifetime. However if a large molecule binds the small molecule, the rotation of the small molecule is slowed and the emitted light remains polarized [18]. The bound and free states of the small molecule are characterized by a polarization or anisotropy value and the strength of the binding interaction can ultimately be determined.

An additional complication could have been that the P1 helix, the helix that is formed by the IGS-tag nucleotide pairs in group I introns, can exist in both a docked and an undocked conformation, at least as shown in the *Tetrahymena* ribozyme [19]. This phenomenon, even if also true for the *Azoarcus* ribozyme, should not alter the within-helix thermodynamics as measured by FA because this technique will produce an average of *K*_D_ values across all conformational states.

#### 2.1.2. Model System

We designed an *Azoarcus* group I intron self-assembly system (Figure 2) to mimic the ca. 155 nucleotide (nt) **WXY** and 45 nt **Z** fragments that are normally recombined by the fully formed 200 nt ribozyme (Figure 1). Here, we were able to systematically vary the middle nucleotide of the IGS and the middle nucleotide of the tag. We chose this nucleotide pair for two reasons. First, the 5′ nucleotide of the IGS is conserved as a G, and the 3′ nucleotide of its complement (the tag) is conserved as a U for normal group I intron splicing, such that a G•U wobble pair immediately prior to the splice site is a strong requisite [20]. Thus we opted not to vary this base pair. Second, our previous studies [4] had focused on the ability of variations in the middle nucleotide of the triplet to affect RNA cooperative network formation, and thus we opted to focus again on this base pair (M•N; Figure 1), saving investigations of the third option for a later study.

The reaction junction where **WXY** is covalently catalyzed to **Z** was designed in the form of a duplex where interaction dynamics are affected as little as possible (Figure 2). The **WXY**-**Z** surrogate duplex is much smaller than the reaction substrate in order to provide a polarization difference, however the sequence of nucleotides in the binding region are the same as in the **WXY** and **Z** junction. This junction is followed by a stem where C and G nucleotides were selectively added to maintain the stability of the duplex molecule at reaction conditions and the fluorophore 6-FAM (6-carboxyfluorescein) was added to the 5′ end of the **Z** fragment. Finally, in the modified system the 3′ end of the tag sequence (U of CNU) was intentionally 3′ dehydroxylated to ensure that there was no nucleophile, and therefore *trans*-esterification was not possible. With this modified scheme, the binding strength between the middle nucleotides of the internal guide sequence and tag could be determined for all 16 possible pairwise combinations.

### 2.2. Catalytic Abilities of the 16 Genotypes

The abilities of all 16 _GMG_**WXY**_CNU_ genotypes to self assemble into **WXYZ** ribozymes when provided with equimolar **Z** were measured previously [5]. These data were aquired in 100 mM MgCl_2_ at pH 7.5 and 48 °C, and were reported as autocatalytic rate constants, which is a non-Michaelis-Menten measure of the dynamics of the recombination reaction at very early time points [16,21]. These values are reproduced in Table 1, alongside the Δ*G*˚ values for IGS-tag triplet binding (in isolation, not in the context of the entire ribozyme-duplex) as predicted by the Turner rules [22].

### 2.3. FA Measurements of the 16 Genotypes

**Figure A1:**
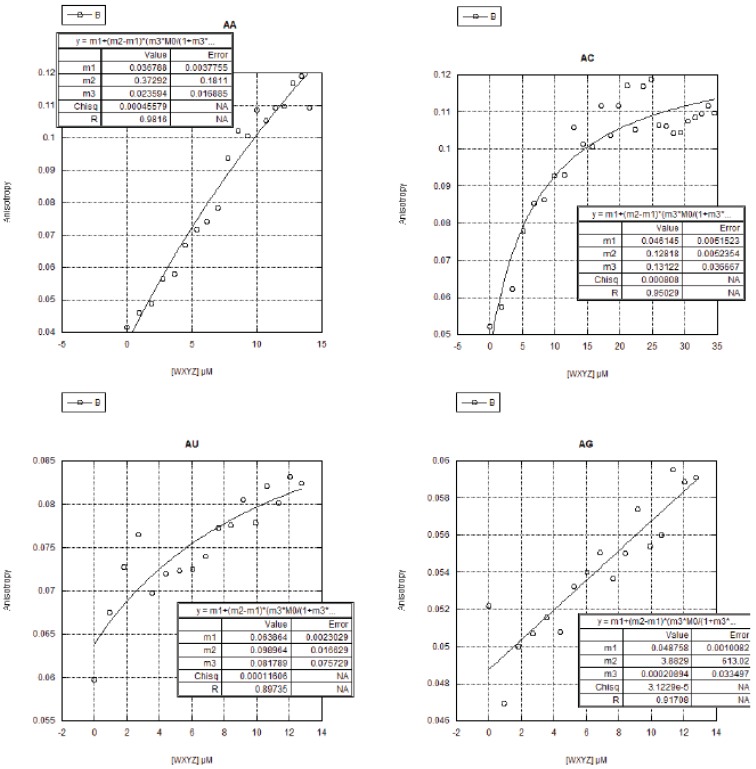
Raw anisotropy data for IGS-tag combinations where the middle nucleotide of the IGS is adenosine. All of these plots show effective saturation in polarization as RNA concentration increases.

**Figure A2:**
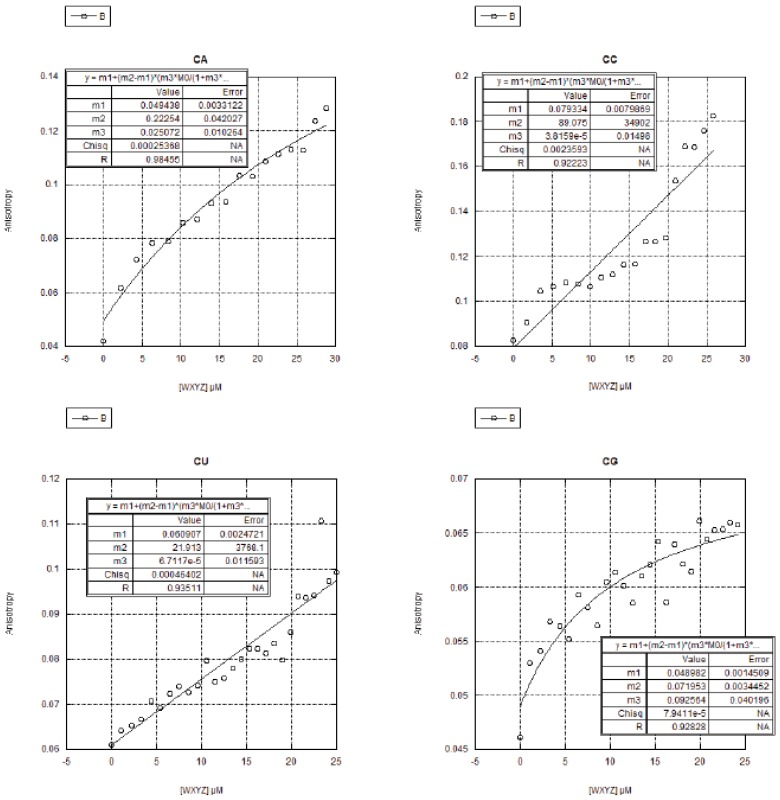
Raw anisotropy data for IGS-tag combinations where the middle nucleotide of the IGS is C. All of these plots show a saturation in polarization as RNA concentration increases.

**Figure A3:**
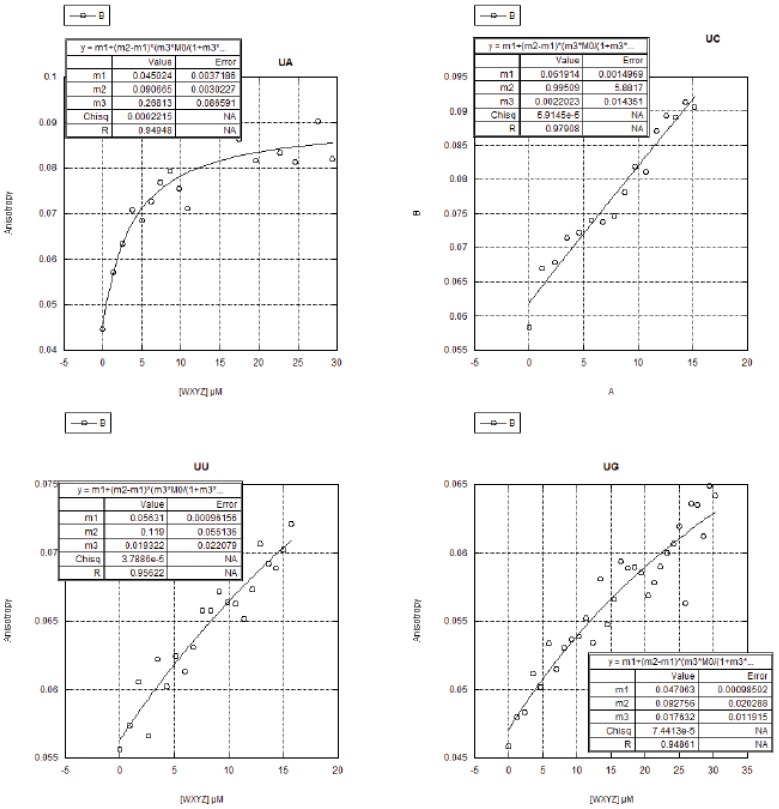
Raw anisotropy data for IGS-tag combinations where the middle nucleotide of the IGS is U. All of these plots show a saturation in polarization as RNA concentration increases.

**Figure A4:**
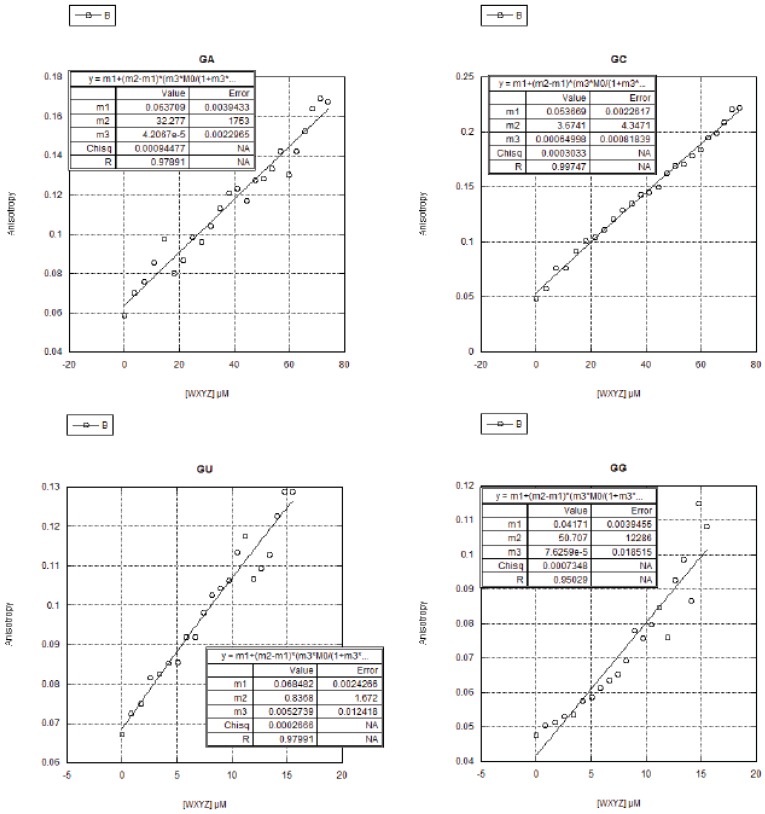
Raw anisotropy data for IGS-tag combinations where the middle nucleotide of the IGS is G. None of these plots show a saturation in polarization as RNA concentration increases, indicating an RNA-RNA aggregation phenomenon for ribozymes with the GGG IGS (see text).

A summary of the *K*_D_ values as measured by the FA experiments is given in the rightmost column of Table 1. The raw FA binding curves from which these data were derived are given in Appendix A, Figure A1, Figure A2, Figure A3 and Figure A4.

#### 2.3.1. Curve Fitting

These plots reflect the increase in polarization as concentration of ribozyme of a particular genotype increases. Ideally, a leveling-off should be seen in which polarization ceases to increase significantly while concentration is still being increased. This leveling-off would indicate a saturation of the duplex, meaning that every duplex molecule is bound to a ribozyme by the IGS-tag binding interaction. Visual examination of Figure A1, Figure A2, Figure A3 and Figure A4 reveals that an asymptote is not reached in some cases, especially for the weaker IGS-tag combinations such as A•G. For poor interactions, this is not unexpected as these results are extrapolated using the binding model formula described in the Materials and Methods (q.v.). To observe an apparent leveling off in such cases one would need to utilize RNA concentrations >100 μM, which is not experimentally viable. Naturally, the error in such cases will be greater than when leveling off occurs below 50 μM. However, in the pool of Watson-Crick pairs and moderately good binders, an asymptote is reached as expected and the correlation follows, and the trends discussed below would hold even in consideration of such error.

Specifically, for genotypes in which the middle nucleotide of the IGS (i.e., M) was A, C, or U, the aniosotropy values (polarization values) approached saturation as the concentration of the ribozyme increased, as expected. However, for ribozymes in which M = G, no saturation was observed, despite attempts to increase ribozyme concentrations well beyond 20 μM, up to 80 μM, which was the highest concentration that could be achieved. We hypothesized that this result was an artifact of RNA multiplexing at high Mg^2+^ concentrations as a consequence of GGG in the IGS forming G-dependent complexes. This was confirmed by native gel electrophoresis (infra vida).

#### 2.3.2. Trends

In general, the Watson-Crick pairs displayed the tightest binding (lowest *K*_D_ values) along with the A•C pair, which is known to be isosteric with the Watson-Crick pairs [23]. Interestingly, the in vivo triplet that forms the isoleucine anticodon, CAU, displays only the fourth strongest catatlytic ability yet has the strongest binding, providing our first hint that triplet binding strength alone cannot be the determinant of the specificity of this interaction. As expected, the non-Watson-Crick pairs show substantially lower binding affinities, the best of these being about 3-fold weaker than the weakest of the strongest six pairs. 

**Figure 3:**
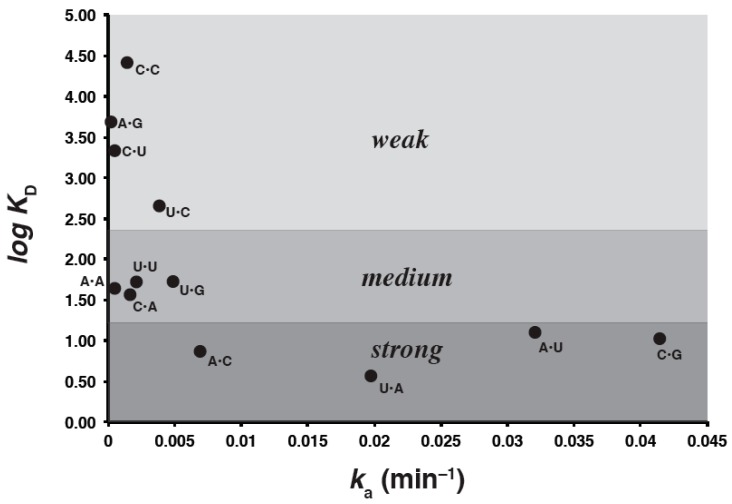
Plot of log (measured binding strength) as a function of autocatalytic rate constant. The untransformed values of these two parameters are only weakly negatively correlated (*r*^2^ = 0.74) but the rank orders are significant (*p* = 0.0057; Spearman’s rank-order correlation test). Visually, three discrete bins of IGS-tag binding strength can be observed.

Excluding the four duplexes in which M = G, we correlated the remaining 12 *K*_D_ values with the autocatalytic self-assembly rate constants obtained previously [5]. In order to quantitatively determine whether there is a statistically significant correlation between the experimental rate constants and equilibrium constants of the genotype combinations, we performed a two-tailed Spearman’s rank-order correlation test. A significant positive association between the *k*_a_ and the *K*_D_ values was detected, with a *p* value of 0.0057. However, the absolute value of the correlation coefficient for the numerical values of these two parameters, when regressed against each other, is only 0.74. While this value is indeed signficant for the rank orders, it does indicate that triplet binding strength is only one component of catalytic efficacy for ribozyme self-assembly. This suggestion is borne out by the predicted Δ*G*˚ (48 °C) values for the triplet–triplet base pairs in isolation (Table 1). Here, only the strongest two Watson-Crick pairs (C•G and G•C) in the middle position of the IGS-tag triplet would be predicted to be stable at 48 °C in 100 mM MgCl_2_ should these trinucleotides be considered alone in solution. The other two Watson-Crick pairs (U•A and A•U), plus the wobble pair U•G should be the next most stable, as expected, followed by every other pair. Yet some of the “poor” catalysts, particularly G•G and A•G (the slowest catalyst), also give Δ*G*˚ values of the same general order as the U•G pair. This suggests some idiosyncratic trends in the contribution of binding to catalysis, while preserving the general result that Watson-Crick pairs have the strongest binding contribution, followed by U•G, followed by all the others. Three main binding-strength categories can be visualized in a plot of log *K*_D_ vs. *k*_a_ (Figure 3).

### 2.4. Native Gel Electrophoresis

**Figure 4:**
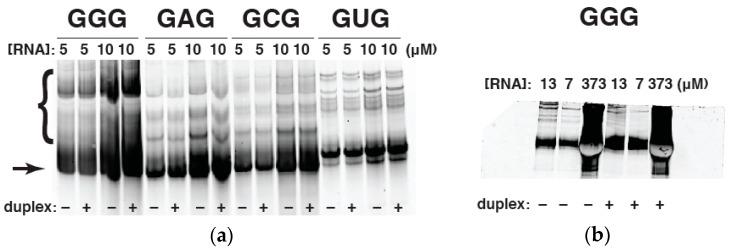
Native-gel electrophoresis of **WXYZ** RNA genotypes. (**a**) All four GMG **WYXZ** RNA genotypes run at either 5 μM or 10 μM concentrations, with or without the complementary duplex RNA present. The upper bands, indicated by bracket, denote excessive multi-RNA complex formation by the GGG genotype. The lower band, indicated by arrow, denotes the dominant (non-aggregated) RNA species; (**b**) The GGG **WXYZ** genotype run in a range of concentrations ranging from 7–373 μM; the gel loading order was chosen to juxtapose maximum and minimum concentrations. The presence of the duplex RNA does not appear to affect aggregation, but the RNA concentration does. All concentrations are given in units of micromolar.

To test our suspicion that excessive complexation of **WXY** RNA genotypes with M = G is preventing a leveling off of polarization at high concentrations, we ran all four **WXY** RNA genotypes on a native polyacrylamide gel (one with no urea denaturant and with 5 mM MgCl_2_ in the running buffer). Significant aggregation can be seen with the GGG genotype; the percentage of slow-migrating RNA species for the GGG, GAG, GCG, and GUG RNAs is 37%, 13%, 15%, and 10%, respectively (Figure 4a). And while the aggregation does not depend on the presence of the duplex, it does become exacerbated as the concentration of the GGG **WXY** RNA increases (Figure 4b). Thus the **WXY** RNA fragment with the GGG IGS does tend to nonspecifically aggregate with itself at higher concentrations, perhaps as a consequence of G-quadruplex formation. Accordingly we deemed that this genotype was not suitable for FA experiments, which require measurements of RNA behavior at high concentrations. However RNA self-assembly experiments, performed at lower RNA concentrations in the 0.1–2.0 μM range [4,5], would not be affected. We also decreased the concentration of MgCl_2_ to 5 mM, and this does appear to reduce the aggregation phenomenon, as would be expected. While, these conditions are not those under which the self-assembly experiments were reported [5], they would tend to minimize polyG interactions.

## 3. Discussion

Here we have demonstrated a significant contribution of triplet nucleotide binding strength to the catalytic rates of ribozyme self-assembly in the *Azoarcus* RNA recombination system. All possible genotype combinations between a group I intron with the IGS = GMG and a duplex with the tag = CNU were observed using fluorescence anisotropy to determine the equilibrium constants of dissociation (*K*_D_). A modified reaction was designed to mimic the recombination reactions of the *Azoarcus* group I intron, where the intron molecule can assemble itself from four fragments using recombination reactions in an autocatalytic fashion such that an assembled ribozyme can catalyze more recombination reactions. The experimentally determined equilibrium constants were found to be weakly but significantly correlated to the rate constants for each respective genotype combination by Spearman’s rank order correlation test.

This research has provided evidence for the hypothesis that there is a correlation between genotype combination and catalytic activity with the thermodynamic strength of the bond. It was shown that in general, canonical Watson-Crick combinations exhibit a lower *K*_D_ than intermediate combinations, which in turn exhibit a lower *K*_D_ values than the catalytically slowest genotype combinations. We can conclude that, while there is a significant correlation between the predicted rate constants of the recombination reaction and the dissociation constants of the modified reaction, several discrepancies in the correlation allude to higher-order molecular interactions occurring. Long-range tertiary interactions in the ribozyme that reshape the local environment of the IGS are the most likely source of this complexity [12,15,24].

Our data underscore the ability of single nucleotide pairs, and in fact single hydrogen-bonding moieties in such pairs, to influence the rates of ribozyme self-assembly in small networks. While the canonical Watson-Crick pairs in the middle position of the IGS-tag triplet dominate both in terms of catalysis and binding, other pairs such as G•U, U•G, and perhaps even G•G have the potential to exert a significant influence on how coalitions of RNA molecules form. Thus the patterns of RNA-RNA group interactions that we quantified previously—such as cooperation, selfishness, dominance, and counter-dominance—ultimately can have a chemical basis at the level of single bonds in aqueous solution. While our data indicate that binding is in fact a substantial factor in these dynamics, other factors such as tertiary interactions and RNA-RNA aggregation can also influence network dynamics. Although three nucleotide pairs provide only 6 or 7 hydrogen bonds, each of which contributing only about 1.9 kcal/mol to duplex stabilization, alteration of single such bonds can provide usable information to nascent evolving systems. It is even conceivable that three is somewhat of a minimal evolving unit in terms of prebiotic nucleic acids, given that within the context of three nucleotide pairs, single hydrogen bond alterations can be detected in an evolutionary context. This thermodynamic “sweet-spot” could even have been the causal determinant in the ultimate selection of three-nucleotide codons.

## 4. Materials and Methods

Short RNA molecules, including those in the duplexes, were purchased from Tri-Link (San Diego, CA, USA). Longer RNAs, i.e., the **WXYZ** ribozymes, were prepared by run-off transcription of DNA templates as described previously [3]. All chemicals were purchased in the highest purity grade possible from Sigma (St. Louis, MO, USA).

To create the duplexes, the **WXY** and **Z** surrogate RNAs were annealed by heating the fragments in solution together to 90 °C, and then allowing them to cool to 20 °C over the course of several hours. All FA experiments were performed at 48 °C. The reaction buffer used in these FA experiments was made from a 5× reaction buffer stock made of 500 mM MgCl_2_ and 150 mM EPPS at pH 7.5, and was diluted to 1× as needed for experiments. These reaction conditions were chosen to match the conditions under which the in vitro self-assembly recombination reactions were observed [3,5]. Fluorescence anisotropy experiments were performed on a PerkinElmer L5 SS Fluorescence Spectrometer (Waltham, MA, USA) using FL Win Lab, with the temperature maintained by a PE Temperature Programmer. For each run, 150 μL 1× reaction buffer was dispensed into cuvette, allowed to heat, and 1 μL duplex was added. Ribozyme (**WXYZ**) was then added in 1–2 μL increments, with at least four polarization values recorded for each addition, the average of which is used for graphing results. Binding plots were made with Kaleidagraph software (v4.5.2, Synergy, Reading, PA, USA).

From the determined average polarization value (P), for each 1–2 μL addition of ribozyme, the anisotropy value (A) can be determined from the formula A = (2P)/(3 − P). The anisotropy value is plotted against ribozyme concentration to generate binding plots, using the formula y = m1 + (m2 − m1) × (m3 × M0/(1 + m3 × M0)); where m1 = anisotropy value when the concentration of ribozyme is zero, m2 = estimated maximum anisotropy, m3 is an estimate of what the *K*_A_ will be for the particular genotype combination, and M0 is the total ribozyme concentration. The *K*_D_ values were obtained by 1/*K*_A_. Figure A1, Figure A2, Figure A3 and Figure A4 show binding plot curves for all 16 genotype combinations, plus a G•C experiment with reduced (5 mM) MgCl_2_ reaction buffer.

Gel electrophoresis was performed using 8% polyacrylamide in 25 cm in a vertical electrophoresis apparatus (CBS Scientific, San Diego, CA, USA). Native gels were run with a reaction buffer containing a 20-fold reduction in MgCl_2_ concentration compared to the FA experiments (5 mM), to observe whether this reduction would reduce or stop aggregation. Gels were run for 4 h at 48 °C and RNA was visualized after staining in SyberGreen II RNA stain (Invitrogen) by a Typhoon Trio + phosphorimager (GE Healthcare, Schenectady, NY, USA), and bands were quantified using Image Quant software (v3, GE Healthcare).

## Figures and Tables

**Table 1:** Catalytic and duplex binding data for all 16 **WXY** genotypes.

Genotype ^a^	Self-Assembly Rates (*k*_a_) ^d^	Δ*G*˚_48_	*K*_D_ (μM) from FA Data Herein ^f^
C•G	0.0415	–0.89 ^e^	10.8
A•U	0.0319	0.97	12.2
U•A ^b^	0.0197	0.77	3.73
G•C	0.0125	–1.4	*1.54 × 10^3^*
G•U	0.0091	3.5	*1.89 × 10^2^*
A•C	0.0069	5.5	7.62
U•G	0.0049	2.6	56.7
U•C	0.0038	5.5	4.54 × 10^2^
U•U	0.0022	5.4	51.8
C•A	0.0020	4.8	39.9
C•C	0.0016	5.5	2.62 × 10^4^
G•G	0.0006	2.8	*1.31 × 10^4^*
G•A	0.0005	5.7	*2.38 × 10^4^*
A•A	0.0004	4.9	42.4
C•U	0.0004	5.3	2.16 × 10^3^
A•G	0.0001	4.0	4.79 × 10^3^
G•C ^c^	ND	ND	63.6

^a^ Abbreviation contains just M and N nucleotides. ^b^ Denotes the wild-type combination found in vivo. ^c^ Data acquired in 5 mM MgCl_2_. ^d^ Autocatalytic rate constants, in units of min^−1^, from ref. [5]. ^e^ Predicted using the Turner rules for the 5′GMG3′–5′CNU3′ triplet in isolation, units of kcal/mol. ^f^ Values in italics obtained from **WXY** genotypes with M = G were excluded from correlation analysis (see Discussion).
